# Assessment of the Impact of Ionizing Radiation Absorption on the Structural, Mechanical and Biophysical Properties of Textiles Used in Multilayer Space Suit

**DOI:** 10.3390/ma15144992

**Published:** 2022-07-18

**Authors:** Izabella Krucińska, Ewa Skrzetuska, Adam K. Puszkarz, Monika Kawełczyk

**Affiliations:** Lodz University of Technology, Faculty of Material Technologies and Textile Design, Institute of Material Science of Textiles and Polymer Composites, 116 Żeromskiego Street, 90-924 Lodz, Poland; izabella.krucinska@p.lodz.pl (I.K.); ewa.skrzetuska@p.lodz.pl (E.S.); 216390@edu.p.lodz.pl (M.K.)

**Keywords:** biophysical comfort, thermal insulation, heat transfer, mass transfer, protective clothing, woven fabric, nonwoven fabric, composite, β-radiation, γ-radiation, CAD modeling, simulation

## Abstract

The article presents research on ergonomics, biophysical comfort and safety of protective clothing. The resistance of the structural, thermal and mechanical properties of five fabrics (CBXS400, GG200T, Twaron CT736, Dyneema HB26 and T1790C), differing in geometry and raw material composition used in space suits, to dangerous ionizing radiation (β and γ) occurring in space was tested. For both types of radiation, four identical one-time doses in the range of 25–100 kGy were used. The effect of the applied absorbed doses of β and γ radiation on the parameters of textiles influencing ergonomics and safety of the cosmonaut’s work was verified by structural tests (micro-computed tomography and optical microcopy), thermal resistance tests (sweating guarded-hotplate) and strength tests (tensile testing machine). Experimental studies of thermal properties are supplemented with heat transport simulations using the finite volume method performed with 3D models of real textiles. The greatest reduction of thermal resistance for Twaron CT736 (−0.0667 m^2^·°C·W^−1^ for 100 kGy of β-radiation) and Dyneema HB26 (−0.0347 m^2^·°C·W^−1^ for 50 kGy of β-radiation) is observed. Strength tests have shown that all tested textiles are resistant to both types of radiation. Three textiles were selected to create a three-layer assembly with potential application in a cosmonaut’s glove (Extravehicular Activity—EVA).

## 1. Introduction

The dynamically developing space industry is stimulated by many reasons, such as curiosity and the desire to discover unknown areas of the universe (such as comets, asteroids, planets, stars, black holes, nebulae and galaxies), expanding knowledge about areas already partially known, searching for a new place to live for human life and the search for unknown civilizations. Over the last decades, scientists from around the world have been improving equipment (spacecrafts, planetary rovers, probes, satellites, telescopes, spectroscopes and others) in order to improve their properties that affect the reliability of their operation in extreme operating conditions as well as the accuracy and reliability of information collected by these devices [1,2,3,4,5,6,7,8]. 

An equally important element of the cosmonaut’s equipment is space suit. The structure of the space suit is adapted to its purpose, there are suits used inside the spacecraft and those used only for space walks—EVA (Extravehicular Activity) [9]. After leaving spacecraft, astronauts stay in extremely difficult environmental conditions, in which they are exposed to numerous dangerous factors such as: very low pressure (up to 10^−16^ hPa), extreme temperatures (from +180 °C in direct sunlight to −173 °C in the shade), solar radiation (ultraviolet, visible, infrared), ionized galactic cosmic radiation, GCR (nuclear component consists of 87% protons, 12% α-radiation, and 1% heavier nuclei, β-radiation, neutrons and γ-radiation), Van Allen radiation belt, atomic oxygen in low Earth orbit (LEO), impacts of micrometeoroids and cosmic debris [10,11,12]. The radiation penetrates the human body, gives back energy and causes ionization of its molecules. The same absorbed dose from different types of radiation may cause a different biological effect in the irradiated organism. For example, α-radiation acts 20 times more intensively on human body than γ-radiation and β-radiation [13]. The harmful biological effect of absorbed radiation on the cosmonaut’s body (e.g., acute bone marrow syndrome, permanent infertility in women and skin necrosis) also depends on other factors like distribution of the dose over time (whether it was a single dose or a dose spread over time), size and type of the area of the body exposed to radiation, age and sex of the irradiated person, body weight and others [14]. According to [5], a one-month absorbed effective dose in space (cosmic radiation) is approximately 10 mSv (which for β and γ radiation corresponds to the absorbed dose 10 mGy.

Over the last decades, scientists from around the world have been improving space suits in order to improve their properties that affect the safety and comfort of the cosmonaut’s work [10,11,15,16,17,18,19,20]. Space suits consist of many layers made of different materials. These layers have different roles and, therefore, have different geometrical and physical parameters. Designing an EVA space suit is a technologically advanced task and requires the use of appropriate textiles that ensure maximum work safety and wearer comfort. The cosmonaut’s work (and therefore moving) in outer space requires high precision. This is not an easy task as the EVA suit is filled with high pressure to simulate the earth pressure. Consequently, the suit behaves like a rigid multilayer balloon and the precise control of the cosmonaut’s movements is therefore very limited [21].

For example, the design of the STS EMU (Extravehicular Mobility Unit) glove requires the use of materials to maintain: the optimal operating temperature of the cosmonaut form 10 °C to 38 °C, provide resistance against abrasion and provide better grip and protect the cosmonaut from injuries.

Meta aramid (e.g., Nomex) is one of the most durable materials used in the production of space suits, as it belongs to the group of flame-retardant fabrics. By subjecting Nomex to the felting process, its properties include strength and thermal properties; they increase, making it suitable for extracellular works. Nomex consists of a long chain of aramid bonds and aromatic rings. Due to the meta orientation in phenyl forms, this material has lower chain stiffness, which leads to greater flexibility while maintaining very high high-temperature properties, similar to para-aramid (e.g., Kevlar), thus retaining greater flexibility than Kevlar [22].

Polytetrafluoroethylene (PTFE, e.g., Teflon) is used in space suits due to its very low friction coefficient and high resistance to extreme temperatures, making it a great insulator. In addition, it is resistant to ignition and has a high resistance to chemicals. The low mechanical properties of Teflon are often improved by adding fillers [23]. Ready-to-use, high strength silicone rubber adhesive sealant (e.g., RTV 157) increases the grip of the glove, but also provides thermal resistance. Polyester film (e.g., Mylar) is characterized by high strength properties, high heat resistance and high thermal insulation properties. When building a space suit, Mylar is the outermost layer of the suit that reflects heat radiation [24].

The aim of the research described in this article is to test the resistance of selected biophysical and mechanical properties of the five textiles with potential use in a multilayer space suit to β radiation and γ radiation. Four identical one-time absorbed doses were used for both types of radiation (25–100 kGy). The study of biophysical properties was supplemented with CAD simulations performed on 3D models of real textiles using the finite volume method.

Tested textiles are made of carbon fibers, para-aramid fibers, ultra-high molecular weight polyethylene (UHMWPE) fibers and glass fibers. Carbon fibers consist almost exclusively of stretched carbon structures. They are characterized by very high resistance to abrasion, corrosion, stretching and creep. These fibers have high dimensional stability and are resistant to sudden changes in temperature, which is especially important when used in a space environment. Carbon fibers do not melt, they sublimate at 3500 °C. They do not lose their properties in non-oxygenated atmospheres, even at the temperature of 2000 °C [25].

Para-aramid fibers are characterized by high mechanical strength, high dimensional stability, low thermal expansion, excellent thermal stability over a wide temperature range, low thermal conductivity, high chemical resistance and low flammability. Aramid fibers are an electrical insulator and have a long service life under static and dynamic loads [26].

UHMWPE fibers have very high mechanical properties and have high energy absorption. Due to these properties, they are often used in the production of bulletproof vests, helmets and boat hulls [27]. The unusual properties of this material result from the molecular structure, and the polyethylene forms very long chains [28]. Moreover, there are no amide groups, hydroxyl groups or aromatic rings in their chains. Consequently, the fibers obtained are resistant to aggressive chemicals, water, moisture, UV radiation and microorganisms. UHMWPE fibers, due to their high abrasion and wear resistance, are also used as a construction material. Polyethylene fibers are known to be highly flexible, although they have a high Young’s modulus. The melting point is between 144–152 °C. The brittle point is minus 150 °C. The upper limit of the temperature at which a fiber can work is approximately 100 °C. It is possible to briefly expose the material to higher temperatures without losing its properties [28].

Glass fibers have low elongation and very high modulus of elasticity. They are often used as reinforcement in polymers. The mechanical strength of the composite largely depends on the length of the fiber used; the longer the fiber, the better the strength. In addition to the length, the diameter of the fiber should also be taken into account; it also has a significant impact on the mechanical properties [29].

The novelty of the presented work is research on the influence of large absorbed doses of cosmic radiation (much higher than the doses critical for human health and life) on mechanical, thermal and structural properties of textiles with potential use in one of the most important elements of multi-layer protective clothing (glove) used by cosmonauts during a walk in outer space. In their works, the authors decided to verify whether and to what extent cosmic radiation may affect the performance of individual components that make up part of the space suits. So far, no studies have been conducted to determine the influence of cosmic rays on thermal and structural properties. The authors compared the effect of different doses of radiation on the functional properties of selected materials, which are most often used in cosmonaut costumes, from the point of view of performance. A cosmonaut’s glove is one of the most demanding parts of a space suit. When designing this part of the suit, safety, ergonomics and the comfort of the astronaut’s work should be taken into account. There must be adequate pressure inside the space glove as in the entire suit, and layers of protective materials that make up the glove give it a very rigid structure. For this reason, the glove greatly limits the mobility of astronauts’ hands, but also leads to many injuries when working outside the spacecraft. Between 2002 and 2004, as many as 47% of 352 symptoms reported by cosmonauts concerned hand injuries. The creation of a comfortable, and at the same time safe, space glove is currently one of the most important design challenges [21]. For this purpose, the authors tried to determine which of the currently used materials will be characterized by the best strength properties and at the same time provide the highest usable and sensory comfort. Contrary to other studies, where the authors focus on the development of new composite systems, e.g., in the form of 3D structures or reinforced with special fibers, they decided to investigate the influence of cosmic rays on their properties [30,31].

## 2. Materials and Methods

### 2.1. Materials

The following five textiles with potential use in multilayer protective clothing were selected for testing:

- The fabric is made of two layers of carbon fibers arranged at an angle of 90°. The layers are made of carbon fibers arranged in one direction. Both layers have been sewn through—CBXS400 (made by Selcom Multiaxial Technology, Fregona, Italy),
- The carbon fabric is made of carbon fiber by unidirectional, smooth weaving—GG200T (made by G. Angeloni, Quarto d’Altino, Italy),
- The fabric is made of para-aramid yarn in a weaving process—Twaron CT736 (made by Teijin Aramid, Arnhem, The Netherlands),
- The fabric made of ultra-high molecular weight polyethylene yarn consists of two single layers of unidirectional sheets glued together at an angle of 90° and consolidated with a polyurethane-based matrix. The fibers are arranged unidirectionally, parallel to each other, they are not woven—Dyneema HB26 (made by Dyneema B.V., Urmond, The Netherlands) and,
- Glass fiber material was used to create a glass mat with shortly chopped glass fibers—T1790 (made by Freudenberg Vliestoffe SE & Co., Weinheim, Germany). The characteristics of the materials are shown in Table 1. Layer thickness, *d*, was determined according to PN-EN ISO 5084:1999 [32], surface mass, *m*, according to PN EN 12127:2000 [33], while total porosity, *P*, and yarn porosity were calculated using X-ray micro-computed tomography.

The CBXS400 and GG200T woven fabrics are made of carbon fibers. CBXS400 is made of two layers of carbon fibers arranged at an angle of 90°. The layers are made of carbon fibers arranged in one direction. Both layers have been sewn. The carbon fabric GG200T is made of carbon fiber by unidirectional, smooth weaving. These fibers characterized by very high resistance to abrasion, corrosion, stretching and creep. These fibers have high dimensional stability and are resistant to sudden changes in temperature, which is especially important when used in a space environment. Twaron CT736 is para-aramid is a woven fabric constructed of para-aramid yarn with high mechanical strength, high dimensional stability, low thermal expansion, excellent thermal stability over a wide temperature range, low thermal conductivity, high chemical resistance and low flammability. DSM Dyneema HB26 is an ultra-high molecular weight polyethylene. Dyneema has very high mechanical properties—it is 15 times stronger than steel. T1790C glass fibers were used to create a glass mat with short-cut glass fibers.

**Figure 1:**
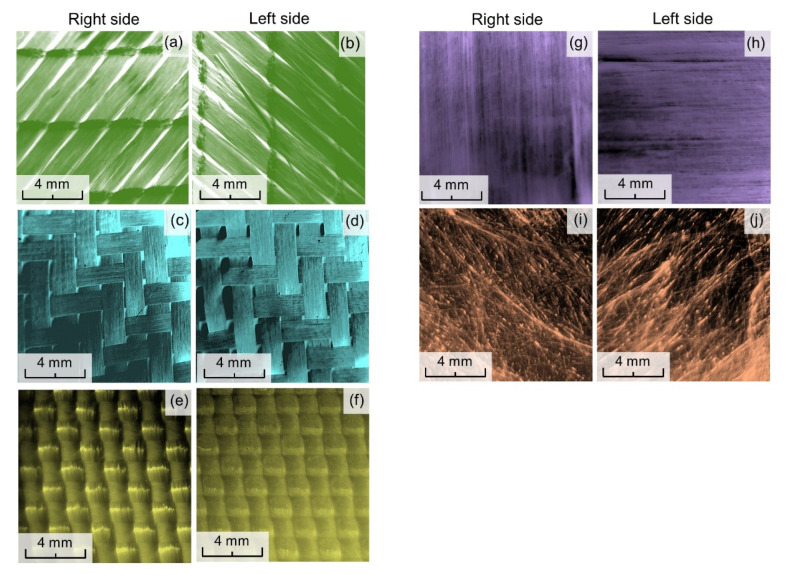
Optical microscopy images of both sides of tested textiles: CBXS400 (**a**,**b**), GG200T (**c**,**d**), Twaron CT736 (**e**,**f**), Dyneema HB26 (**g**,**h**), T1790C (**i**,**j**).

Optical microcopy images of both sides of the surface tested textiles (Figure 1) were obtained using optical microscope (PZO, Warsaw, Poland) equipped with a digital optical camera (DLT-Cam PRO, Delta Optical, Warsaw, Poland) and software (DLT CamViewer, version 3.7, Delta Optical, Warsaw, Poland).

From the images, one can identify a different weave for woven fabrics: (GG200T—2 × 2 twill, Twaron CT736—basket 2 × 2). The CBXS400 woven fabric is made of two layers in which the continuous fibers are oriented parallel to each other. These layers are connected by a polyester thread, and the fibers systems in both layers are perpendicular to each other (Figure 1a,b). Dyneema HB26 is an ultra-high molecular weight polyethylene (UHMWPE) fiber based composite laminate. The material consists of four single layers of unidirectional sheet cross plied at 90 degrees to each other and consolidated with a polyurethane (PUR) based matrix (Figure 1g,h). In the case of Twaron CT736, the weave is also visible from the laminate side, because the polyamide foil is transparent (Figure 1f). T1790 nonwoven fabric composed of random oriented C-glass fibers (Figure 1i,j).

**Figure 2:**
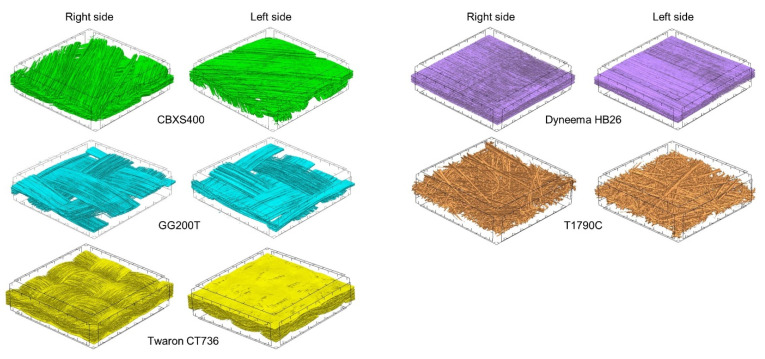
Micro-computed tomography 3D reconstructions of both sides of tested textiles (surface area: 4 mm × 4 mm).

Three-dimensional reconstruction of the tested textiles (Figure 2) was made using X-ray micro-computed tomography (SkyScan 1272; Bruker, Kontich, Belgium), and is presented in Figure 2. Micro-computed tomography (Micro-CT) images were obtained applying the following scanning conditions: X-ray source voltage 50 kV, X-ray source current 200 µA, pixel size 4.5 µm. A 180° rotation was performed with a rotation step of 0.2° and no filter was selected. The reconstructions show the textiles with a reduced surface area (4 mm × 4 mm). Based on the 3D reconstructions, the geometrical parameters were used to design three-dimensional models of real textiles, on the basis of which heat transfer simulations were performed to characterize their thermal insulation properties. A more detailed description of modeling is provided in Section 2.2.2.

### 2.2. Methods

#### 2.2.1. Modification Methods

##### β Radiation

**Figure 3:**
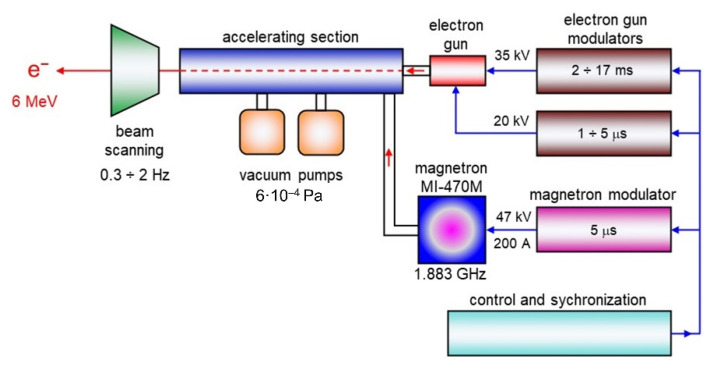
Scheme of the Linac ELU-6e accelerator.

Modification of tested textiles with b radiation was performed with the use of Linac ELU-6e linear accelerator (Electronica Company, Moscow, Russia) [34] the scheme of which is shown in Figure 3. For the modification, a stream of electrons with an energy of 6 MeV was used. The textiles were irradiated with the doses: 25 kGy, 50 kGy, 75 kGy and 100 kGy.

##### γ Radiation

**Figure 4:**
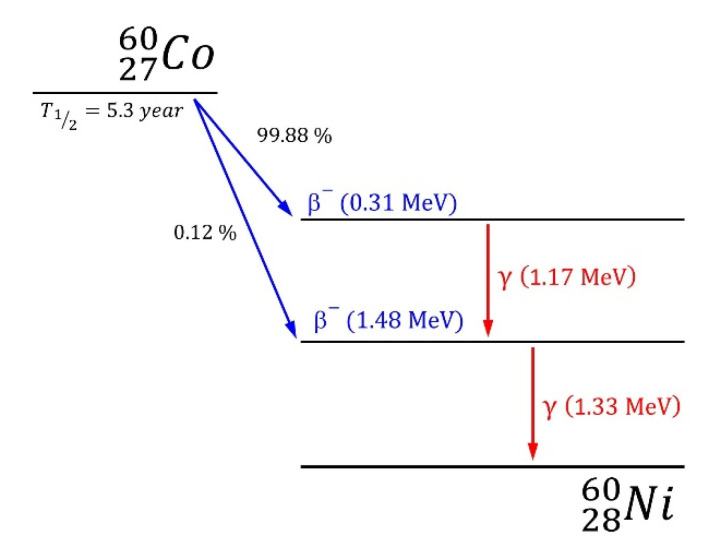
Decay scheme of ^60^Co.

Modification of tested textiles with γ radiation was performed using the source of gamma radiation ^60^Co [35]. Radioactive cobalt isotope ^60^Co decays by emitting an electron (β^−^ decay) with a half-life of 5.3 years into an excited state of ^60^Ni, which then decays immediately to the ground state of ^60^Ni, via two gamma decays of energies: 1.17 MeV and 1.33 MeV (Figure 4). The textiles were irradiated with the doses: 25 kGy, 50 kGy, 75 kGy and 100 kGy.

#### 2.2.2. Modeling

##### Model Designing

**Figure 5:**
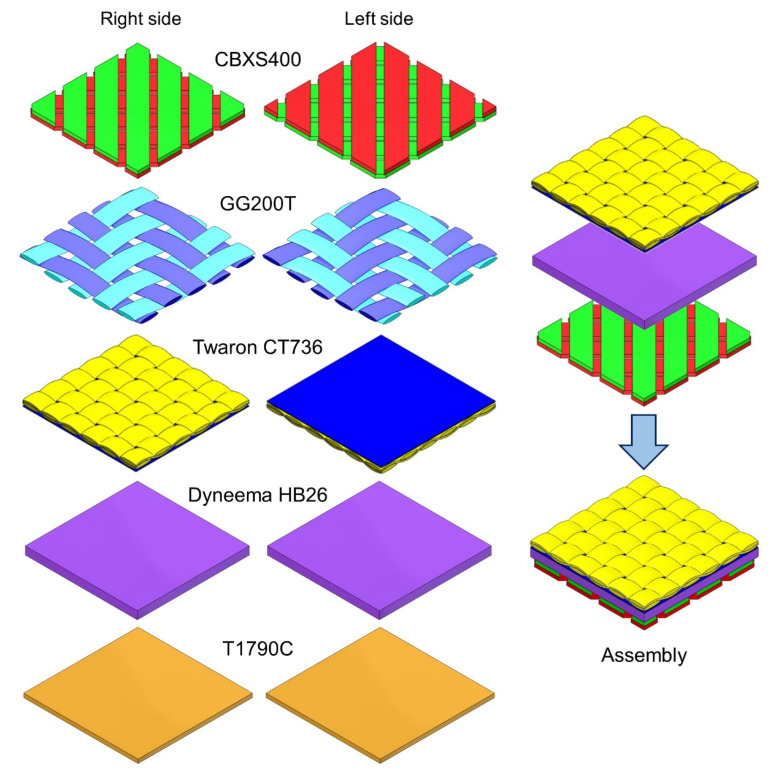
Both side view of 3D models of five tested textiles and assembly (surface area: 10 mm × 10 mm).

Based on measurements of geometric parameters of tested textiles using computed microtomography, the three-dimensional geometric models and assembly model built from CBXS400, Dyneema HB26 and Twaron CT736 (composed of the most suitable materials for packaging, which include all raw materials except glass fiber), using SolidWorks 2014 CAD software (Dassault Systèmes, Waltham, MA, USA) [36], were designed (Figure 5). The following calculated parameters of the textiles have been taken into account when designing the textile models: layer thickness and total porosity. In addition, yarn thickness, yarn shape and cross-sectional area, yarn porosity, distance between the weft yarns and distance between the warp yarns were mapped for three woven fabrics (CBXS400, GG200T and Twaron CT736). The yarn in all woven fabrics models was designed as a monofilament (without taking into account the individual fibers and the spaces between them filled with air). Polyamide foil from Twaron CT736, composite Dyneema HB26 and nonwoven fabric T1790C and yarn in three woven fabrics (CBXS400, GG200T and Twaron CT736), due to their complicated internal structure, were mapped as the homogenized 3D objects with physical parameters (density, specific heat and thermal conductivity coefficient) resulting from porosity presented in Table 1. The model of the assembly (built from CBXS400, Dyneema HB26 and Twaron CT736) does not take into account individual threads connecting layers due to its negligible low weight and negligibly low influence on heat transfer inside the assembly. The physical parameters of raw materials necessary for the simulation [37,38,39] were assigned to the geometric models (Table 2).

##### Simulations of Heat Transfer

Physical basis

Heat transfer simulations inside 3D models of tested textiles assembles were carried out using by SolidWorks Flow Simulation 2014 (Dassault Systèmes, Waltham, MA, USA) software and the finite volume method. The method allows prediction of fluid flow using equations of energy conservation and equations of Navier–Stokes [39]. A wider specification of applied computational method has been presented in previous articles on heat and mass transport modeling in textiles [37,38,40,41,42,43,44,45].

Initial conditions of simulations

**Figure 6:**
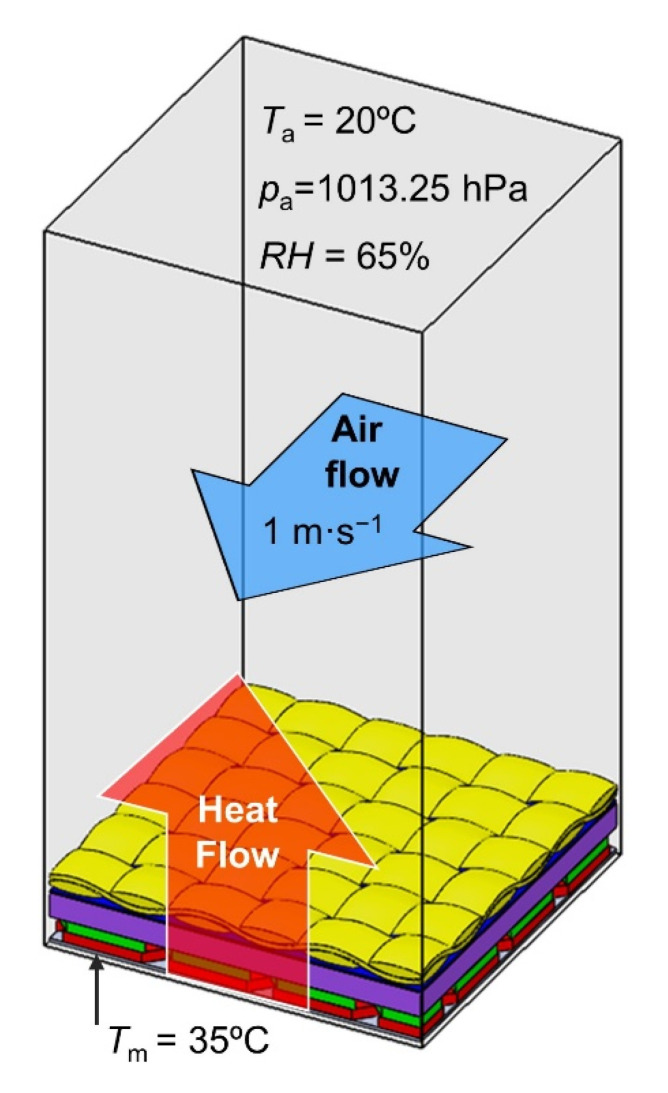
Conditions of simulations inside computational domain with dimensions of 10 mm × 10 mm × 20 mm.

The initial environmental conditions for heat transfer modeling through textiles corresponded to the initial conditions in which the thermal resistance tests were carried out. Each model of the five textiles and assembly model were placed on a sweating guarded hotplate model with a constant temperature of 35 °C. The textiles model and hotplate model were placed inside a rectangular computational domain 20 mm high filled by air of following parameters *T*_a_ = 20 °C, *p*_a_ = 1013.25 hPa, *RH* = 65%. Moreover, an air stream of 1 was present over the textile model. Moreover, a horizontal air stream of 1 m·s^−1^ was present above the model (Figure 6). As a result of the temperature difference between the hotplate and the surroundings, a heat transfer occurs through the textile model in a vertical upward direction. As a result of heat transfer, successive layers of the textile model heat up, reaching a certain constant temperature after reaching a steady state. As a result of the heat transfer modeling, the temperature difference between the top side and bottom side of each textile model was calculated (temperature drop, *D*_T_ [°C]) in steady state.

To imitate an infinite textile model and hotplate model propagating outside of computational domain in all four horizontal directions, periodic boundary conditions were assumed. Computational domain was divided into three types of cells: solid (containing hotplate or/and textile), gas (air) and partial (plate and air or textile and air). Number of cells was different for each textile model due to its spatial geometry (Table 3).

#### 2.2.3. Evaluation of Modified Textiles Properties

##### Thermal Resistance (Sweating Guarded-Hotplate Test)

**Figure 7:**
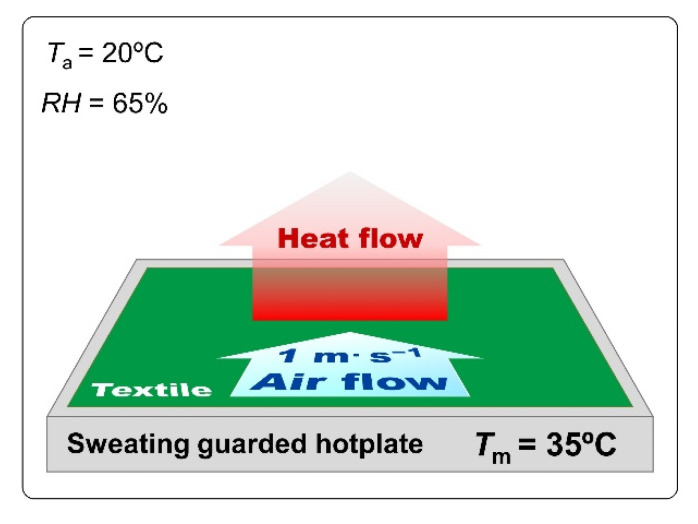
Conditions of textiles thermal resistance measurement.

Evaluation of the thermal resistance of the textiles were performed using a Sweating Guarded Hotplate 8.2 (made by Measurement Technology Northwest in Seattle, DC, USA) according to PN-EN ISO 1192:2014-11 [46]. The parameter related to the evaluation of the thermal resistance is calculated according to the following formula:

$$R_{\mathrm{ct}}=\frac{\left(T_{m}-T_{a}\right)\cdot A}{H-\Delta H_{c}}-R_{\mathrm{ct}0}$$

where: *T*_m_—sweating guarded hotplate temperature [°C]; *T*_a_—air temperature [°C]; *A*—surface of the measuring plate [m^2^]; *H*—heating power supplied to the measuring plate [W]; ∆*H*_c_—heating power correction in case of measuring thermal resistance [W]; and *R*_ct0_—instrument constant for measuring thermal resistance, [m^2^·°C·W^−1^]. The thermal resistance tests were performed consecutively under the following conditions: *T*_a_ = 20 °C, *RH* = 65%, air flow speed 1 m·s^−1^ (Figure 7).

##### Maximum Force and Elongation at Maximum Force (Tensile Testing Machine)

Maximum force (*F*) and elongation at maximum force (*L*) of tested textiles were measured using tensile testing machine (Instron, model 4204 made in Norwood, MA, USA). Breaking force and elongation at break for tested textiles were determined according to PN-EN ISO 13934-1:2013 [47]. The size textile samples were 250 mm × 50 mm. The tests were performed at crosshead speed of 100 mm·min^−1^. The measurements of both mechanical parameters were made in two directions: transverse direction orientation (*F*_⊥_, *L*_⊥_) and machine direction orientation (*F*_‖_, *L*_‖_).

## 3. Results

**Figure 8:**
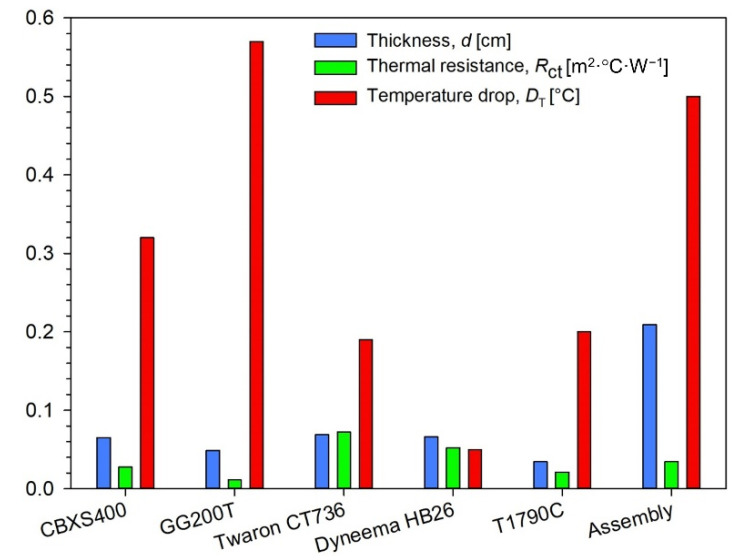
Comparison textile thickness, thermal resistance and simulated temperature drop.

The results of heat transfer simulations performed on 3D models of tested five textiles and assembly (built from CBXS400, Dyneema HB26 and Twaron CT736) show the influence of the geometry and the raw material composition of the assembly on its thermal insulation properties. Analyzing the results presented in Table 4 and Figure 8, it can be concluded that the best heat insulator is the woven fabric GG200T, while the Dyneema HB26 composite is the worst insulating. Similar values of temperature drop were observed for the Twaron CT736 laminated fabric and the T1790C non-woven fabric which clearly differed in the total porosity and thickness.

**Figure 9:**
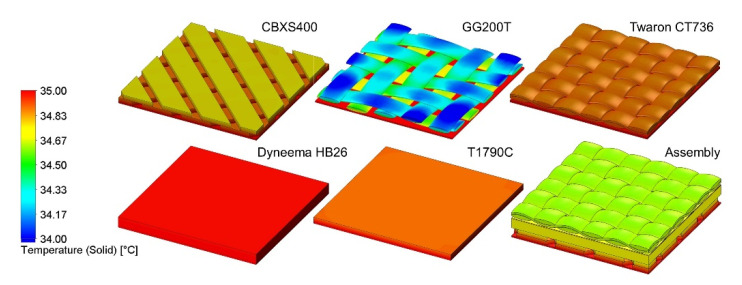
Temperature distributions on 3D models of five tested textiles and assembly.

Temperature distributions on 3D models of tested five textiles and the assembly were presented in Figure 9. In the case of CBXS400, Twaron CT736, Dyneema HB26, T1790C and assembly the entire volume of the model, a uniform temperature drop can be seen with the distance from the hotplate. For these models, the temperature is the same over the entire top surface. In the case of the GG200T woven fabric one can observe heterogeneous temperature distribution due to the large spaces between the weft and warp yarns and the asymmetrical 2 × 2 twill weave and it is possible to observe areas with different temperatures located at the same distance from the hotplate.

**Figure 10:**
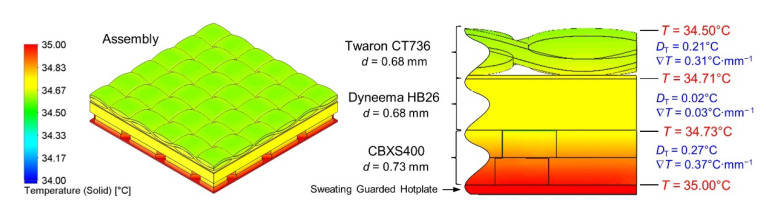
Temperature distributions on 3D model of assembly.

Figure 10 additionally shows the temperature distribution in a cross section of the assembly. The calculated temperatures at the boundary of the layers of the three textiles (CBXS400, Dyneema HB26 and Twaron CT736) making up the assembly are marked. On the basis of these temperatures, the temperature drops *D*_T_ [°C] and the temperature gradients ∇*T* [°C·mm^−1^] on each thickness *d* of the three layers were calculated.

From the results of the assembly heat transport modeling shown in Figure 10, it can be seen that that the Dyneema HB26 composite has a tenfold lower *D*_T_ and a tenfold lower ∇*T* compared to the Twaron CT736 laminated fabric, although the fabrics are of equal *d*. Moreover, similar values of *D*_T_ and ∇*T* for Twaron CT736 and CBXS400 were observed.

**Figure 11:**
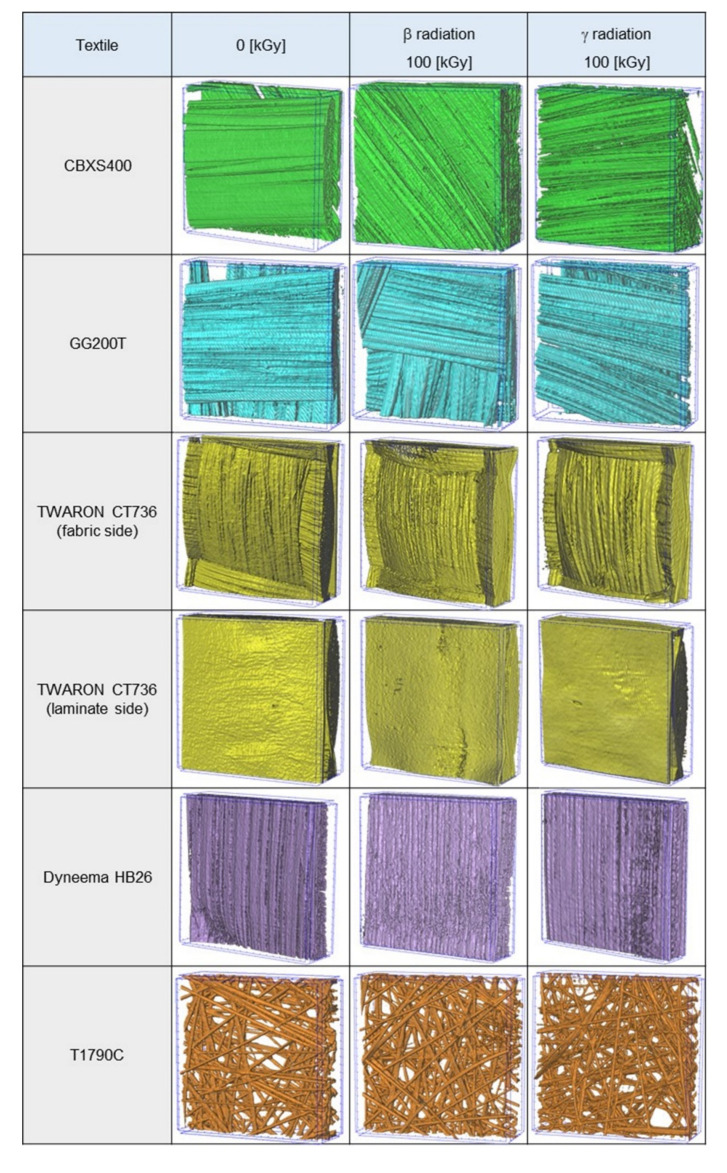
Three-dimensional micro-computed tomography reconstructions of unmodified and irradiated textiles (surface area 2 mm × 2 mm).

To observe potential micro changes in the structure (on the surface and inside) of irradiated textiles, three-dimensional reconstructions of the textiles were performed using computer microtomography (Figure 11). The reconstructions show of tested textiles (in case of Twaron CT736 on both sides) with a reduced surface area (2 mm × 2 mm) unmodified and exposed to maximum absorbed dose (100 kGy) of β-radiation and γ-radiation. On the basis of the performed observations and measurements of the obtained reconstructions, no changes were found in any of the tested textiles.

In Table 5 and Table 6, structural, biophysical and mechanical parameters of unmodified and modified textiles (respectively by β radiation and γ radiation) were presented. The obtained results show that both β radiation and γ radiation used in the same doses affect changes in thickness (*d*), surface mass (*m*) and thermal resistance (*R*_ct_), as well as the maximum force (*F***_⊥_** and *F*_‖_) and elongation at maximum force (*L***_⊥_** and *L*_‖_) of all tested textiles.

**Figure 12:**
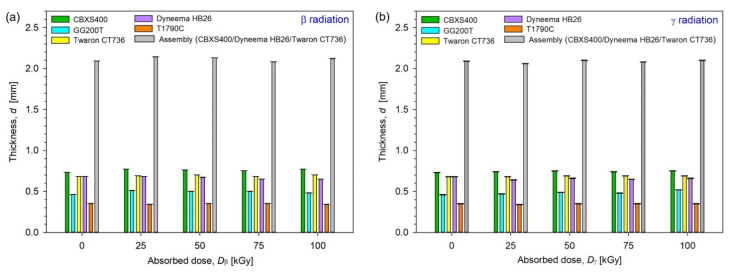
Thickness of unmodified and irradiated textiles using (**a**) β-radiation, (**b**) γ-radiation.

By analyzing the test results presented in Figure 12 and in Table 5 and Table 6, it can be noticed that the maximum thickness of the tested materials is characterized by the carbon fiber fabrics (CBXS400), which is approximately 0.75 mm. Composite of polyethylene fibers (Dyneema HB26) and laminated fabric made of para-aramid fibers (Twaron CT736) materials have equally high thickness measurement results. These values are, respectively, 0.66 mm and 0.69 mm. When analyzing the results, one can see no or minimal changes in the thickness of the tested samples after exposure to both sources of radiation. In the case of irradiation with γ carbon fiber fabrics (CBXS400), the smallest changes occur for the doses of 25 kGy and 75 kGy (+0.01 mm), while the greatest changes were observed in the case of carbon fiber fabrics (GG200T) irradiated with γ for the dose of 100 kGy (+0.06 mm).

In the case of β-irradiation with laminated fabric made of para-aramid fibers (Twaron CT736), there were no changes for the dose of 75 kGy (0.00 mm), similarly for glass fiber nonwovens (T1790C) for the doses of 50 kGy and 75 kGy (0.00 mm), while the greatest changes were observed for the dose of 25 kGy (−0.04 mm) for composite of polyethylene fibers (Dyneema HB26).

For assembly (built from CBXS400, Dyneema HB26 and Twaron CT736) the smallest *d* changes of β-irradiated textile compared to unmodified ones (2.09 mm) occur for dose 75 kGy (−0.01 mm), while the biggest changes occur for the dose 25 kGy (+0.05 mm). In the case of γ-irradiated assembly the smallest changes occur for dose 75 kGy (−0.01 mm), while the biggest changes have been observed for the dose 25 kGy (−0.03 mm).

**Figure 13:**
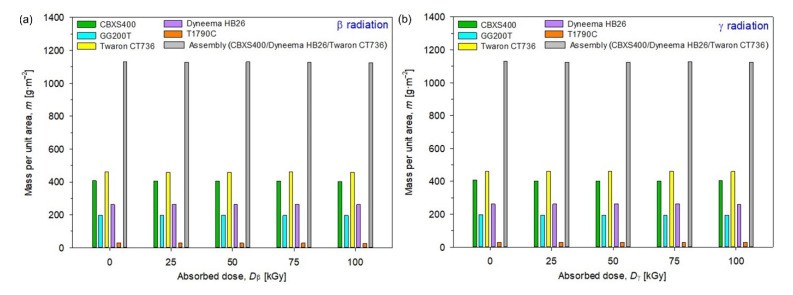
Mass per unit area of unmodified and irradiated textiles using (**a**) β-radiation, (**b**) γ-radiation.

In Figure 13 mass per unit area (*m*) of unmodified and modified by β radiation (left plot) and γ radiation (right plot) textiles were presented.

By analyzing the test results presented in Figure 13, Table 5 and Table 6, it can be observed that the most resistant textiles to radiation in terms of conservation of mass were Twaron CT736 and T1790C, for which the smallest changes in the area mass per unit area *m* compared to unmodified textiles were observed. For Twaron CT736, the biggest changes occur for the dose 100 kGy of β-radiation (−0.99 g·m^−2^), while for γ-irradiated Twaron CT736, the biggest changes have been observed for the dose 75 kGy (+1.81 g·m^−2^). For T1790C, the biggest changes occur for the dose 100 kGy of β-radiation (−1.50 g·m^−2^) and for the dose 100 kGy of γ-radiation (−0.16 g·m^−2^).

For assembly (built from CBXS400, Dyneema HB26 and Twaron CT736), the smallest *m* changes of β-irradiated textile compared to unmodified ones (1129.44 g·m^−2^) occur for dose 75 kGy (−0.30 g·m^−2^), while the biggest changes occur for the dose 100 kGy (−5.34 g·m^−2^). In the case of γ-irradiated assembly, the smallest changes occur for dose 75 kGy (−2.14 g·m^−2^), while the biggest changes have been observed for the dose 50 kGy (−4.30 g·m^−2^).

**Figure 14:**
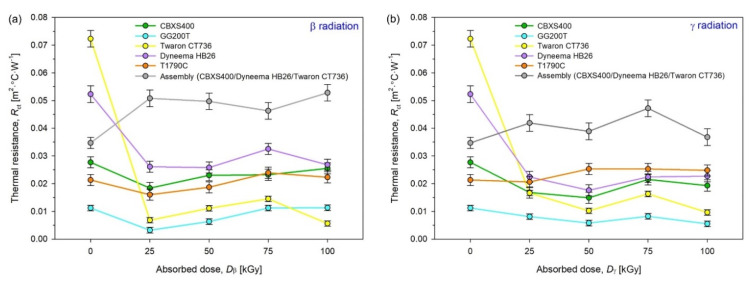
Thermal resistance of unmodified and irradiated textiles using (**a**) β-radiation, (**b**) γ-radiation (lines guide the eye).

In Figure 14, thermal resistance (*R*_ct_) of unmodified and modified by β radiation (left plot) and γ radiation (right plot) textiles were presented.

By analyzing the test results presented in Figure 14, Table 5 and Table 6, it can be observed that the most resistant textiles to radiation in terms of thermal insulation were GG200T and T1790C, for which the smallest changes in the thermal resistance *R*_ct_ compared to unmodified textiles were observed. For GG200T, the biggest changes occur for the dose 25 kGy of β-radiation (−0.0080 m^2^·°C·W^−1^), while for γ-irradiated GG200T, the biggest changes have been observed for the dose 75 kGy (−0.0030 m^2^·°C·W^−1^). For T1790C, the biggest changes occur for the dose 25 kGy of β-radiation (−0.0050 m^2^·°C·W^−1^) and for the doses 50 kGy and 75 kGy of γ-radiation (−0.0040 m^2^·°C·W^−1^).

For assembly (built from CBXS400, Dyneema HB26 and Twaron CT736), the smallest *R*_ct_ changes of β-irradiated textile compared to unmodified ones (0.0347 m^2^·°C·W^−1^) occur for dose 75 kGy (−0.0116 m^2^·°C·W^−1^), while the biggest changes occur for the dose 100 kGy (−0.0181 m^2^·°C·W^−1^). In the case of γ-irradiated assembly, the smallest changes occur for dose 100 kGy (−0.0021 m^2^·°C·W^−1^), while the biggest changes have been observed for the dose 75 kGy (−0.0125 m^2^·°C·W^−1^).

**Figure 15:**
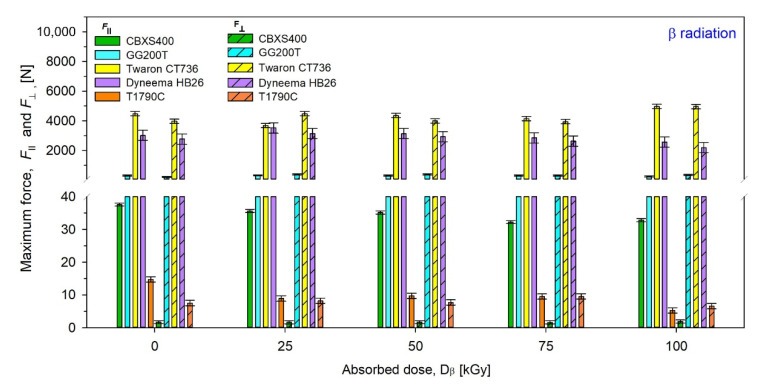
Maximum force of unmodified and β irradiated textiles.

Results of maximum force (*F***_⊥_** and *F*_‖_ ) of unmodified and modified by β radiation were presented in Figure 15, while those by γ radiation were presented in Figure 15.

**Figure 16:**
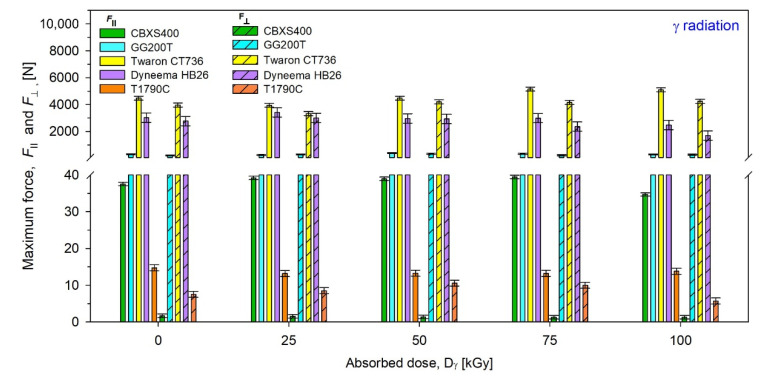
Maximum force of unmodified and γ-irradiated textiles.

By analyzing the test results presented in Figure 15 and Figure 16 and Table 5 and Table 6, it can be inferred the most resistant textiles to radiation in terms of tensile force were CBXS400 and GG200T, for which the smallest changes in the maximum force *F* compared to unmodified woven fabrics were observed. For CBXS400, the biggest *F***_⊥_** changes occur for the dose 100 kGy of β-radiation (−0.23 N) and the biggest *F*_‖_ changes occur for the dose 75 kGy of β-radiation (−5.32 N), while for γ-irradiated CBXS400, the biggest *F***_⊥_** changes have been observed for the dose 75 kGy (−0.38 N) and the biggest *F*_‖_ changes have been observed for the dose 100 kGy (−2.85 N). For GG200T, the biggest *F***_⊥_** changes occur for the dose 25 kGy of β-radiation (+168.95 N) and the biggest *F*_‖_ changes occur for the dose 100 kGy of β-radiation (−65.7 N), while for γ-irradiated GG200T, the biggest *F***_⊥_** changes have been observed for the dose 50 kGy (+120.83 N) and the biggest *F*_‖_ changes have been observed for the dose 100 kGy (+83.24 N).

**Figure 17:**
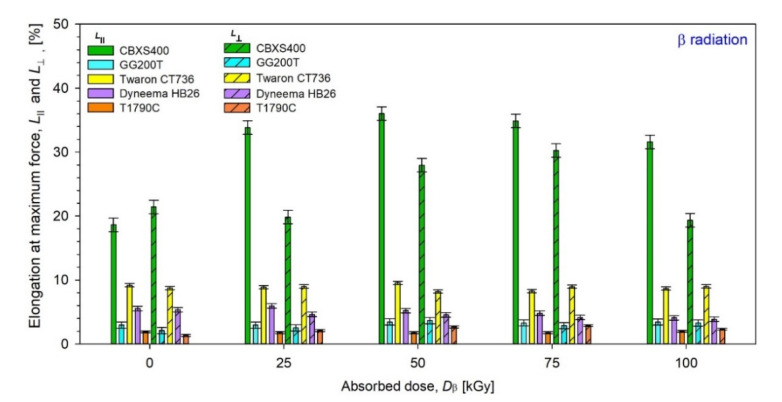
Elongation at maximum force of unmodified and β irradiated textiles.

**Figure 18:**
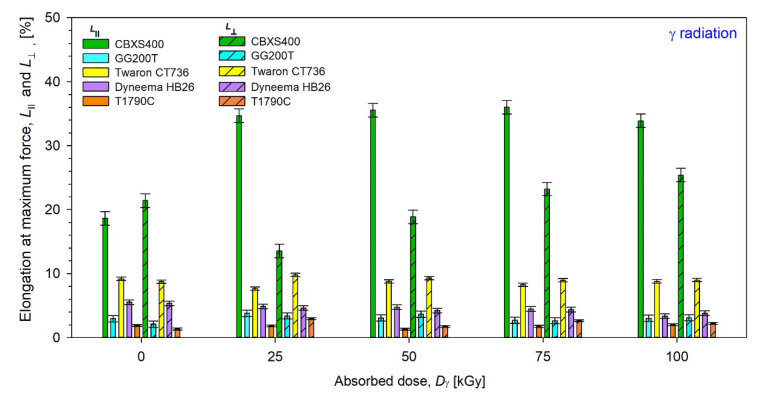
Elongation at maximum force of unmodified and γ-irradiated textiles.

Results of elongation at maximum force (*L***_⊥_** and *L*_‖_) of unmodified and modified by β radiation were presented in Figure 17, while those by γ radiation were presented in Figure 18.

By analyzing the test results presented in Figure 17 and Figure 18, Table 5 and Table 6, it can be inferred the most resistant textiles to radiation in terms of mechanical strength were Twaron CT736, for which the smallest changes in the elongation at maximum force *L* compared to unmodified textile were observed.

For Twaron CT736, the biggest *L* **_⊥_** changes occur for the dose 100 kGy of β-radiation (−0.53%) and the biggest *L*_‖_ changes occur for the dose 75 kGy of β-radiation (−0.93%), while for γ-irradiated Twaron CT736 the biggest *L* **_⊥_** changes have been observed for the dose 25 kGy (+1.06%) and the biggest *L*_‖_ changes have been observed for the dose 25 kGy (−1.53%).

## 4. Discussion

The paper presents temperature drops in the thickness of the layer of the tested products. The Dyneema HB26 textile model is characterized by the smallest temperature drops between the thickness of the material, this value is equal to 0.05 °C. Twaron CT736—0.19 °C and T1790C—0.20 °C have slightly higher values. The highest values of temperature drops were calculated for the carbon fiber fabrics CBXS400 and GG200T materials, which were, respectively, 0.32 ℃ and 0.57 °C. Dyneema HB26 composite is characterized by high porosity resulting from the arrangement of the fibers. This material consists of two single layers of unidirectional sheets glued together at an angle of 90° and consolidated with a polyurethane-based matrix. This arrangement of the fibers causes the porosity to develop in the various directions of the material. The high porosity of the T1790C glass nonwoven fabric is related to the three-dimensional arrangement of the fibers. In the models of carbon woven fabrics (CBXS400 and GG200T), the pores appear perpendicular to the plate model. The smaller the temperature drop, the greater the conductivity of the textile material. In the case of the tested materials, the simulated temperature drops do not exceed 0.6 °C. Changes in the thermal insulation properties of the tested materials depend on the orientation of pores in the material, thickness and thermal conductivity of the tested materials.

Twaron CT736 is characterized by the highest mass per unit area, which is due to the presence of the PA laminate. The CBXS400 carbon woven fabric achieves an equally high mass per unit area. The lowest mass per unit area values were obtained by T1790C made of glass fiber—the value is approximately 29 g·m^−2^. When analyzing the test results, it can be noticed that there are no significant mass per unit area changes of the tested samples before and after exposure to both types of radiation.

The CBXS400 carbon woven fabric is characterized by the maximum thickness among the tested materials, which is approximately 0.75 mm. Dyneema HB26 and Twaron CT736 materials have equally high thickness measurement results. These values are 0.66 mm and 0.69 mm, respectively. When analyzing the results, one can see no or minimal thickness changes (within the measurement error limits) of the tested samples after exposure to both sources of radiation.

In the Dyneema HB26 composite, we can observe that under the influence of both types of radiation thermal resistance decreased by approximately 0.03 m^2^·°C·W^−1^. Textiles absorbed γ-radiation have lower thermal resistance than textiles absorbed the same doses β-radiation. It is worth noting that when the textiles are exposed to β-radiation, an increase in the value of thermal resistance at the dose of 75 kGy is visible.

Both in the case of textiles that absorb β and γ-radiation, the Twaron CT736 significantly decreased their thermal resistance and amounts to 0.0723 m^2^·°C·W^−1^ before irradiation. Already after irradiation with the lowest dose of 25 kGy, thermal resistance dropped to 0.0166 m^2^·°C·W^−1^ in the case of gamma radiation and 0.0068 m^2^·°C·W^−1^ in the case of β-radiation. The thermal resistance value of Twaron does not change significantly with the increase of the radiation dose, it fluctuates at the value of 0.01 m^2^·°C·W^−1^.

After irradiating the CBXS400 using β and γ-radiation, one can see a thermal resistance decrease at the dose of 25 kGy. In the case of irradiation of CBXS400 using β-radiation this value gradually increases with increasing doses. The textiles exposed to γ-radiation, in comparison with textiles exposed to γ-radiation, have a lower thermal resistance values.

For GG200T, in the case of exposure to β-radiation, the lowest dose caused a sudden decrease in the thermal resistance value of the material. As the doses increase, the thermal resistance value increases, stabilizing at a value equal to the unmodified GG200T. In the case of γ-radiation, there is a downward trend in the thermal resistance value of the textiles. A slight anomaly is visible at the dose of 75 kGy, but with a higher dose of radiation, a decrease in the value of the material tested is visible.

The thermal resistance of the T1790C nonwoven fabric, after the exposure to both types of radiation, increased. In the case of γ-radiation, at a dose of 50 kGy, the thermal resistance value stabilizes—the resistance value is approximately 0.0253 m^2^·°C·W^−1^. With the highest radiation dose, a minimal decrease in thermal resistance is noticeable.

After irradiating the tested textiles using β-radiation, at the dose of—75 kGy, the materials reach a value similar to that of the textiles exposed to γ-radiation. However, a decrease in the thermal resistance value is noticeable with an increase in the radiation absorbed dose.

The value of the unmodified Twaron CT736—0.0723 m^2^·°C·W^−1^ and unmodified Dyneema HB26—0.0523 m^2^·°C·W^−1^ prove the low thermal conductivity of both textiles. In case of both materials, even a small absorbed dose of radiation leads to a significant decrease in the thermal resistance. In the case of carbon woven fabrics (CBXS400, GG200T) and glass nonwoven fabric (T1790C), absorbed doses of radiation do not have such a drastic effect on the thermal resistance value of the textiles. It is worth noting that thermal resistance of unmodified textiles, however, is small.

When analyzing the results of the strength tests, it can be noticed that in the case of Dyneema HB26, the average value of the strength of the broken textiles decreases with the increase of the absorbed dose of radiation. The mechanical strength of the textiles becomes colder, regardless of the type of radiation or the direction in which the textiles were torn off. In the case of Twaron CT736, a slight increase in strength values is noticeable from the absorbed dose of radiation—25 kGy. When analyzing the values of the textiles exposed to β-radiation, an increase in the value of the torn textiles along with the absorbed dose of radiation is also visible. As for the GG200T carbon woven fabric, it has a very unstable weave. Thus, it is possible to observe numerous deviations of the strength values, which are not related to the types of radiation in use, but to the weave of a given sample. Apart from the weave of the GG200T carbon fabric, it is worth noting the high values obtained when tearing the textiles. The T1790C nonwoven fabric has a very low tensile strength. As the absorbed doses of radiation increase, the properties of the T1790C decrease noticeably. It is especially visible at the highest absorbed doses of radiation—100 kGy.

Analyzing the test results, Dyneema HB26 and Twaron CT736 have the best tear resistance. The CBXS400, GG200T and T1790 C textile materials have much worse results. The test results of these materials are closely related to the type of weave used for their production. In the case of the GG200T carbon woven fabric, the biggest problem is the lack of fabric reinforcement through the use of stitching or laminate. CBXS400 has two layers of carbon fibers arranged at an angle of 90°, reinforced with stitching. In T1790C nonwoven fabric, the fibers are arranged in different directions.

The design of the material system for the construction of the EVA space glove was made on the basis of documents provided by NASA and the tests performed. When analyzing the individual results of the tested fabrics, three materials were used for the final project.

The three-layer assembly was designed with: carbon fiber fabrics (CBXS400), composite of polyethylene fibers (Dyneema HB26) and laminated fabric made of para-aramid fibers (Twaron CT736). These three textiles were characterized by the most stable structure and high strength properties (in the case of CBX400 only in the perpendicular direction). In addition, the carbon woven fabric has been proposed for the layer on the skin side, which is characterized by the greatest flexibility and arrangement, thus ensuring the greatest sensory comfort for the skin. The assembly was subjected to a thermal resistance test. When analyzing the test results, it can be noticed that the use of three materials simultaneously changed the thermal resistance value of the textiles. The combination of CBXS400, Dyneema HB26 and Twaron CT736 materials reduced the thermal conductivity of the assembly. Based on test results for irradiated textiles using β-radiation, there is a noticeable increase in the thermal resistance value of the textiles. In the case of γ-radiation, some deviations are visible, but these values fluctuate around the value of unmodified sample—0.04 m^2^·°C·W^−1^.

**Figure 19:**
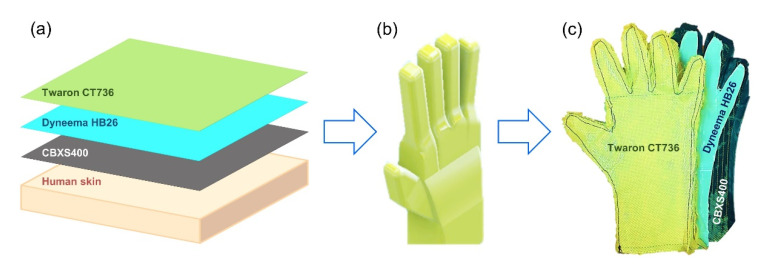
The glove model: (**a**) modeled construction design of the space glove including materials (from the left: Twaron CT736, Dyneema HB26, CBXS400, astronaut’s skin), (**b**) model of the EVA type space glove prototype for cosmonaut, (**c**) photo of the gloves used to construct the EVA type space glove prototype for astronaut.

The glove model was made with Autodesk Inventor software. Figure 19 shows the material model of the space glove. The blue plane marks the astronaut’s skin, the closest to the skin is CBXS400, then Dyneema HB26, and the outer layer is Twaron CT736.

The CBXS400 carbon woven fabric, which has a very high fatigue resistance and high abrasion resistance, was used as the internal material closest to the astronaut’s body. In addition, it is characterized by high thermal resistance. Dyneema HB26 composite, which is made of ultra-high molecular weight raw material, was selected as the middle layer of the assembly. This feature is especially important in outer space due to the ability to trap UV radiation. This material also has a very high absorption of mechanical energy, which is especially important during the impact of micrometeorites in space. Twaron CT736 woven fabric was used as the outer material. This material is characterized by very high strength and thermal properties, thanks to which it will constitute the main barrier against the conditions of space that are hostile to humans.

## 5. Conclusions

Analyzing the properties of composite of polyethylene fibers (Dyneema HB26), laminated fabric made of para-aramid fibers (Twaron CT736), carbon fiber fabrics (CBXS400), carbon fiber fabrics (GG200T) and glass fiber nonwovens (T1790C), and taking into account the results of the research, three textiles were selected, on the basis of which, the prototype of the space glove (EVA type) was created using CBXS400, Dyneema HB26, Twaron CT736. CBXS400 carbon woven fabric was used as the inner material, closest to the astronaut’s body. Carbon fabric has a very high fatigue resistance and high abrasion resistance. In addition, it is characterized by high thermal resistance. Dyneema HB26 is one of the ultra-high molecular weight materials. This property is especially important in outer space because it can trap UV radiation. This material also has a very high energy absorption, especially important during impacts of micrometeorites in space. Twaron CT736 was used as the outer material. This material is characterized by very high strength and thermal properties, it will be the main barrier against the dangerous environment of space. In addition, it has a low thermal expansion coefficient and high dimensional stability.

The best values for the thermal resistance of test specimens were shown by laminated fabric made of para-aramid fibers (Twaron CT736), composite of polyethylene fibers (Dyneema HB26) and carbon fiber fabrics (CBXS400). Due to the combination of these materials, a system with high values of thermal resistance was obtained. The GG200T carbon fabric is the worst. Determination of the breaking strength allowed to select materials with the best strength properties, including: Dyneema HB26 and Twaron CT736. In the case of testing Dyneema HB26 samples, a slight decrease in strength properties is visible along with an increase in radiation doses. On the other hand, in the case of Twaron CT736, an increase in these values is visible with increasing doses. The worst results were reported for the T1790C. The computed microtomography examination showed the structure of all materials very accurately. The Twaron CT736 was characterized by the best fiber arrangement and no cavities. Dyneema HB26 was also characterized by a very good arrangement of fibers in the material. The worst structure can be seen when observing the GG200T carbon cloth. Due to its unstable weave, this material was characterized by large fiber shifts and losses. When analyzing the simulation in the SolidWorks program, the highest results of the temperature drop in the thickness of the layer of the tested textiles were calculated for the GG200T carbon fabric, 0.57 ℃, and CBXS400, 0.32 ℃. Dyneema HB26 has the lowest value, 0.05 ℃.

We also analyzed the weave and the structure of the tested materials. In terms of weave and structure, Twaron CT736 and Dyneema HB26 are the best. Twaron CT736 is a material covered with a laminate on one side, it has high stiffness and durability. Dyneema HB26 consists of two layers of glued fibers. It is characterized by high flexibility while maintaining high strength properties. The T1790C glass mat was characterized by a very brittle structure, low force allowed the material to break. The GG200T textile material had equally poor properties, it consisted of loosely woven carbon fibers. As a result of working on samples, the weave often shifted and the fibers fell out of the weave. The CBXS400 was made of the same fiber; unlike the GG200T, it had stitching that kept two layers of fibers in one configuration.

## Figures and Tables

**Table 1:** Characteristics of five tested textiles.

No	Textile Name	Textile Type	Layer Composition	Layer Thickness ^(a)^ [mm]		Surface Mass ^(b)^ [g·m^−^^2^]	Total Porosity ^(c)^ [%]	Yarn Porosity ^(c)^ [%]
1	CBXS400	woven fabric	carbon fiber	0.73		407.77	54	34
2	GG200T	woven fabric	carbon fiber	0.48		197.01	68	16
3	Twaron CT736	woven fabric	aramid fiber	0.58	0.68	459.96	36	18
		laminate	polyamide foil	0.10			0	-
4	Dyneema HB26	composite	polyethylene	0.68		261.71	62	-
5	T1790C	nonwoven fabric	glass fiber	0.35		28.95	73	-

(a) determined according to PN-EN ISO 5084:1999 [32], (b) determined according to PN EN 12127:2000 [33], (c) determined using X-ray microtomography.

**Table 2:** Physical features of raw materials applied in simulations.

Physical Parameter	Carbon Fiber	Aramid Fiber	PA	PE	Glass Fiber	Air
**density [kg·m^−3^]**	2000	1360	1230	980	2500	1.20
**specific heat [J·kg^−1^·°C^−1^]**	800	1390	2050	1800	840	1005
**thermal conductivity [W·m^−1^·°C^−1^]**	100	0.18	0.22	0.50	0.04	0.03

**Table 3:** Number of cells in 3D models of tested textile models.

3D Model	Solid Cells	Gas Cells	Partial Cells
CBXS400	25,310	22,533	14,548
GG200T	9785	15,105	9125
Twaron CT736	13,982	14,265	9497
Dyneema HB26	4376	6276	2280
T1790C	4376	6676	2280
Assembly	104,862	78,728	51,535

**Table 4:** Comparison textile thickness and simulated temperature drop.

No	Textile Name	Layer Thickness *d* [mm]	Thermal Resistance *R*_ct_ [m^2^·°C·W^−1^]	Temperature Drop *D*_T_ [°C]
1	CBXS400	0.652	0.0277	0.32
2	GG200T	0.489	0.0112	0.57
3	Twaron CT736	0.688	0.0723	0.19
4	Dyneema HB26	0.659	0.0523	0.05
5	T1790C	0.346	0.0213	0.20
6	Assembly	2.090	0.0347	0.50

**Table 5:** Structural, biophysical and mechanical properties of unmodified and β-irradiated textiles.

Textile	β Radiation	Thickness, *d* [mm]	Surface Mass, *m* [g·m^−2^]	Sweating Guarded Hotplate	Tensile Testing Machine			
	Absorbed Dose, *D*_β_ [kGy]			Thermal Resistance, *R*_ct_ [m^2^·°C·W^−1^]	Maximum Force		Elongation at Maximum Force	
					*F*_⊥_ [N]	*F*_‖_ [N]	*L*_⊥_ [%]	*L*_‖_[%]
CBXS400	0	0.73 ± 0.003	407.77	0.0277 ± 0.002	1.65 ± 0.47	35.63 ± 0.45	21.43 ± 1.06	18.63 ± 1.04
	25	0.77 ± 0.003	406.00	0.0184 ± 0.002	1.69 ± 0.52	35.13 ± 0.49	19.85 ± 1.02	33.84 ± 1.14
	50	0.76 ± 0.003	406.53	0.0230 ± 0.002	1.63 ± 0.48	32.24 ± 0.51	27.95 ± 1.15	36.03 ± 1.27
	75	0.75 ± 0.003	405.35	0.0232 ± 0.002	1.89 ± 0.56	32.80 ± 0.43	30.26 ± 1.24	34.88 ± 1.15
	100	0.77 ± 0.003	402.39	0.0255 ± 0.002	1.65 ± 0.43	35.63± 0.48	19.34 ± 1.14	31.61 ± 0.98
GG200T	0	0.46 ± 0.003	197.01	0.0112 ± 0.001	385.62 ± 38.07	312.62 ± 41.15	2.12 ± 0.49	2.97 ± 0.48
	25	0.51 ± 0.003	196.99	0.0032 ± 0.001	379.10 ± 41.11	310.20 ± 37.98	2.54 ± 0.55	2.96 ± 0.52
	50	0.50 ± 0.003	197.81	0.0063 ± 0.001	308.53 ± 39.55	310.62 ± 37.01	3.67 ± 0.51	3.46 ± 0.53
	75	0.50 ± 0.003	196.07	0.0112 ± 0.001	336.96 ± 37.85	239.50 ± 42.05	2.87 ± 0.48	3.30 ± 0.48
	100	0.48 ± 0.003	197.02	0.0113 ± 0.001	385.62 ± 40.01	312.62 ± 43.17	3.28 ± 0.47	3.44 ± 0.51
Twaron CT736	0	0.68 ± 0.003	459.96	0.0723 ± 0.003	4472.16 ± 145.25	3681.84 ± 141.98	8.75 ± 0.27	9.21 ± 0.28
	25	0.69 ± 0.003	459.42	0.0068 ± 0.001	3987.41 ± 151.34	4354.99 ± 153.19	9.00 ± 0.26	8.88 ± 0.27
	50	0.70 ± 0.003	459.15	0.0111 ± 0.001	3938.91 ± 150.16	4139.47 ± 150.46	8.22 ± 0.33	9.55 ± 0.29
	75	0.68 ± 0.003	459.96	0.0145 ± 0.001	4958.64 ± 149.14	4968.85 ± 154.68	8.96 ± 0.31	8.28 ± 0.24
	100	0.70 ± 0.003	458.97	0.0056 ± 0.001	4472.16 ± 146.33	3681.84 ± 148.67	9.01 ± 0.32	8.69 ± 0.26
Dyneema HB26	0	0.68 ± 0.003	261.71	0.0523 ± 0.003	2769.13 ± 348.51	3017.94 ± 356.74	5.35 ± 0.36	5.55 ± 0.37
	25	0.68 ± 0.003	263.26	0.0261 ± 0.002	3131.67 ± 354.65	3512.98 ± 347.43	4.63 ± 0.38	5.96 ± 0.39
	50	0.67 ± 0.003	264.35	0.0258 ± 0.002	2922.84 ± 351.47	3130.32 ± 348.00	4.56 ± 0.35	5.23 ± 0.32
	75	0.65 ± 0.003	263.83	0.0325 ± 0.002	2625.62 ± 340.12	2846.78 ± 356.13	4.16 ± 0.40	4.82 ± 0.41
	100	0.65 ± 0.003	262.74	0.0268 ± 0.002	2185.25 ± 352.68	2559.53 ± 355.01	3.89 ± 0.37	4.09 ± 0.39
T1790C	0	0.35 ± 0.003	28.95	0.0213 ± 0.002	7.52 ± 0.82	14.73 ± 0.81	1.36 ± 0.15	1.89 ± 0.14
	25	0.34 ± 0.003	28.72	0.0160 ± 0.002	8.19 ± 0.79	8.89 ± 0.85	2.11 ± 0.17	1.78 ± 0.13
	50	0.35 ± 0.003	28.63	0.0187 ± 0.002	7.70 ± 0.86	9.72 ± 0.79	2.67 ± 0.14	1.78 ± 0.15
	75	0.35 ± 0.003	28.69	0.0239 ± 0.002	9.53 ± 0.80	9.50 ± 0.77	2.88 ± 0.18	1.77 ± 0.18
	100	0.34 ± 0.003	27.45	0.0223 ± 0.002	6.56 ± 0.78	5.25 ± 0.80	2.31 ± 0.19	1.97 ± 0.16
Assembly	0	2.09 ± 0.003	1129.44	0.0347 ± 0.002	−	−	−	−
	25	2.14 ± 0.003	1128.68	0.0508 ± 0.003	−	−	−	−
	50	2.13 ± 0.003	1130.03	0.0497 ± 0.003	−	−	−	−
	75	2.08 ± 0.003	1129.14	0.0463 ± 0.003	−	−	−	−
	100	2.12 ± 0.003	1124.10	0.0528 ± 0.003	−	−	−	−

**Table 6:** Structural, biophysical and mechanical properties of unmodified and γ-irradiated textiles.

Textile	γ Radiation	Thickness, *d* [mm]	Surface Mass, *m*[g·m^−^^2^]	Sweating Guarded Hotplate	Tensile Testing Machine			
	Absorbed Dose, *D*_γ_ [kGy]			Thermal Resistance *R*_ct_ [m^2^·°C·W^−^^1^]	Maximum Force		Elongation at Maximum Force	
					*F*_⊥_ [N]	*F*_‖_ [N]	*L*_⊥_ [%]	*L*_‖_ [%]
CBXS400	0	0.73 ± 0.003	407.77	0.0277 ± 0.002	1.66 ± 0.47	37.56 ± 0.46	21.43 ± 1.05	18.63 ± 1.15
	25	0.74 ± 0.003	401.96	0.0168 ± 0.002	1.55 ± 0.52	39.27 ± 0.50	13.55 ± 1.07	34.70 ± 1.14
	50	0.75 ± 0.003	401.31	0.0149 ± 0.002	1.39 ± 0.50	38.98 ± 0.51	18.87 ± 1.11	35.55 ± 1.15
	75	0.74 ±0.003	402.26	0.0215 ± 0.002	1.28 ± 0.49	39.51 ± 0.48	23.22 ± 1.21	36.03 ± 1.09
	100	0.75 ± 0.003	405.29	0.0193 ± 0.002	1.29 ± 0.48	34.71 ± 0.42	25.42 ± 1.22	33.89 ± 1.20
GG200T	0	0.46 ± 0.003	197.01	0.0112 ± 0.001	216.67 ± 38.07	305.20 ± 36.77	2.12 ± 0.51	2.97 ± 0.48
	25	0.47 ± 0.003	193.92	0.0081 ± 0.001	280.62 ± 41.05	251.83 ± 39.14	3.39 ± 0.48	3.80 ± 0.56
	50	0.49 ± 0.003	192.82	0.0058 ± 0.001	337.50 ± 40.89	388.44 ± 38.78	3.67 ± 0.47	3.09 ± 0.51
	75	0.48 ± 0.003	194.30	0.0082 ± 0.001	226.39 ± 36.99	333.73 ± 36.47	2.64 ± 0.50	2.71 ± 0.47
	100	0.52 ± 0.003	192.58	0.0055 ± 0.001	269.51 ± 37.85	286.56 ± 42.01	3.13 ± 0.48	3.04 ± 0.48
Twaron CT736	0	0.68 ± 0.003	459.96	0.0723 ± 0.003	3967.28 ± 148.25	4477.13 ± 146.16	8.75 ± 0.30	9.21 ± 0.31
	25	0.68 ± 0.003	461.34	0.0166 ± 0.001	3331.32 ± 147.41	3945.70 ± 152.16	9.81 ± 0.28	7.68 ± 0.30
	50	0.69 ± 0.003	459.53	0.0102 ± 0.001	4201.17 ± 151.02	4481.59 ± 140.03	9.31 ± 0.27	8.79 ± 0.28
	75	0.69 ± 0.003	461.77	0.0163 ± 0.001	4155.68 ± 150.17	5153.9 ± 144.89	8.96 ± 0.26	8.28 ± 0.25
	100	0.69 ± 0.003	459.69	0.0096 ± 0.001	4241.82 ± 147.05	5097.58 ± 143.64	9.01 ± 0.29	8.81 ± 0.26
Dyneema HB26	0	0.68 ± 0.003	261.71	0.0523 ± 0.003	2769.13 ± 348.51	3017.94 ± 354.78	5.35 ± 0.35	5.55 ± 0.36
	25	0.64 ± 0.003	261.84	0.0224 ± 0.002	2998.71 ± 352.01	3418.36 ± 350.14	4.63 ± 0.37	4.89 ± 0.37
	50	0.66 ± 0.003	263.87	0.0176 ± 0.002	2926.91 ± 347.99	2964.10 ± 356.07	4.22 ± 0.38	4.76 ± 0.42
	75	0.65 ± 0.003	263.27	0.0224 ± 0.002	2365.81 ± 340.26	2987.36 ± 347.18	4.39 ± 0.41	4.48 ± 0.40
	100	0.66 ± 0.003	260.74	0.0227 ± 0.002	1691.87 ± 358.00	2480.84 ± 345.62	3.83 ± 0.35	3.36 ± 0.39
T1790C	0	0.35 ± 0.003	28.95	0.0213 ± 0.002	7.52 ± 0.81	14.73 ± 0.78	1.36 ± 0.14	1.89 ± 0.13
	25	0.34 ± 0.003	28.97	0.0206 ± 0.002	8.50 ± 0.83	13.18 ± 0.81	2.97 ± 0.17	1.83 ± 0.16
	50	0.35 ± 0.003	29.00	0.0253 ± 0.002	10.54 ± 0.79	13.24 ± 0.86	1.76 ± 0.17	1.33 ± 0.18
	75	0.35 ± 0.003	29.08	0.0253 ± 0.002	9.97 ± 0.82	13.22 ± 0.81	2.64 ± 0.12	1.77 ± 0.14
	100	0.35 ± 0.003	28.79	0.0248 ± 0.002	5.71 ± 0.85	13.81 ± 0.79	2.22 ± 0.15	2.05 ± 0.12
Assembly	0	2.09 ± 0.003	1129.44	0.0347 ± 0.002	−	−	−	−
	25	2.06 ± 0.003	1125.14	0.0419 ± 0.003	−	−	−	−
	50	2.10 ± 0.003	1124.71	0.0389 ± 0.003	−	−	−	−
	75	2.08 ± 0.003	1127.30	0.0472 ± 0.003	−	−	−	−
	100	2.10 ± 0.003	1125.72	0.0368 ± 0.003	−	−	−	−
